# Physiatrist referral preferences for postacute stroke rehabilitation

**DOI:** 10.1097/MD.0000000000004356

**Published:** 2016-08-19

**Authors:** David J. Cormier, Megan A. Frantz, Ethan Rand, Joel Stein

**Affiliations:** a Department of Rehabilitation and Regenerative Medicine, Columbia University College of Physicians and Surgeons; b Division of Rehabilitation Medicine, Weill Cornell Medical College; c New York-Presbyterian Hospital, New York, NY.

**Keywords:** outcomes, rehabilitation, stroke

## Abstract

This study was intended to determine if there is variation among physiatrists in referral preferences for postacute rehabilitation for stroke patients based on physician demographic characteristics or geography.

A cross-sectional survey study was developed with 5 fictional case vignettes that included information about medical, social, and functional domains. Eighty-six physiatrist residents, fellows, and attendings were asked to select the most appropriate postacute rehabilitation setting and also to rank, by importance, 15 factors influencing the referral decision. Chi-square bivariate analysis was used to analyze the data.

Eighty-six surveys were collected over a 3-day period. Bivariate analysis (using chi-square) showed no statistically significant relationship between any of the demographic variables and poststroke rehabilitation preference for any of the cases. The prognosis for functional outcome and quality of postacute facility had the highest mean influence ratings (8.63 and 8.31, respectively), whereas location of postacute facility and insurance had the lowest mean influence ratings (5.74 and 5.76, respectively).

Physiatrists’ referral preferences did not vary with any identified practitioner variables or geographic region; referral preferences only varied significantly by case.

## 1 Introduction

Nearly 800,000 individuals experience a stroke each year in the United States, at a cost of 33.6 billion dollars.^[1]^ Whereas some persons with stroke recover fully, many are left with substantial disability. Stroke is the leading cause of serious long-term disability in this country.^[1]^ Given the impact on individuals with stroke and the substantial resources devoted to their care, it is important to gain a greater understanding of which poststroke interventions lead to the best outcomes. One area of controversy is the type of rehabilitation facility where persons with stroke should receive their rehabilitative care.

Poststroke rehabilitation options include inpatient rehabilitation facilities (IRFs), skilled nursing facilities (SNFs), long-term acute care hospitals (LTACHs), home therapy, and outpatient therapy. The process of assessing rehabilitation needs and selecting the most appropriate rehabilitation option for a person with acute stroke is complex and not well-studied. Depending on the institution, this determination may be made by nurses, case managers, social workers, physical therapists, occupational therapists, speech and language pathologists, and/or physicians (including physiatrists, neurologists, internists, and others). Physiatrists’ role in this process varies among hospitals, with some hospitals involving physiatry routinely, and others rarely or never. Physiatrists’ role includes the medical and functional assessment as it encompasses all of the rehabilitation needs, through a strong relationship within the interdisciplinary rehabilitation team. Physiatrists are arguably the physicians with the most specific training in stroke rehabilitation, and it is therefore important to better understand their referral preferences for these patients.

Many factors may be considered when determining the most appropriate poststroke rehabilitation option for a given patient. These factors may include the severity and nature of neurological and functional deficits, medical comorbidities, provider and facility relationships, insurance coverage, cost, geographical proximity and location of available facilities, and patient and family preference.^[2,3]^ When referral to an IRF is being considered, the question of whether or not a patient will be able to participate in and benefit from the 3 hours of therapy that are mandated in an IRF is of particular concern.

Assessment protocols are not standardized, and there is little reassurance that patients are reliably receiving the most appropriate rehabilitation. Furthermore, there exist no standardized criteria or guidelines to assist referral teams in predicting which poststroke discharge option is optimal for each patient. To optimize patient outcomes after stroke, more information is needed about which patients benefit most from rehabilitation in each setting. Knowing who is making these referral decisions and how they are making them is an important first step towards reaching this goal.

Given the large number of individuals involved in making decisions regarding rehabilitation level of care, and the many factors that contribute to this decision, it is unsurprising that research has found variation in referral patterns. After stroke, patients are more likely to be evaluated for rehabilitation needs if they are hospitalized in a stroke unit.^[4]^ Measures of activities of daily living (ADLs) ability after stroke are predictive of discharge home versus a rehabilitation institution, but do not distinguish between patients discharged to SNF and patients discharged to IRF.^[5]^

When rehabilitation consultation teams assist in making the referral decision, patient outcomes improve.^[6]^ Ilet et al^[7]^ further found that the likelihood of discharge to a rehabilitation unit is influenced by variation in practice among hospitals. Geographic proximity to an IRF has been shown to be a substantial predictor of the likelihood of discharge to IRF.^[8]^ Variation in the utilization and intensity of poststroke rehabilitation services has also been demonstrated by Medicare beneficiaries’ payment analysis.^[9,10]^

Patients who suffer a stroke benefit from early rehabilitation.^[11,12]^ There is also some indication in the literature that patients admitted to IRF experience better functional recovery than those admitted to SNF.^[13–17]^ To date, studies comparing IRF to SNF outcomes in the United States have all been observational in nature, and no randomized studies have been performed. As a result, comparing IRF to SNF stroke rehabilitation outcomes is complicated by the differences between the patient populations referred for these 2 different types of care. Multiple factors known to influence outcomes after stroke (age, cognition, functional level, continence) have also been found to be different in those receiving postacute stroke rehabilitation in IRFs and those receiving this rehabilitation in SNF.^[6]^

We sought to examine postacute stroke rehabilitation referral preferences among physiatrists. We hypothesized that there is variation among physiatrists in referral preferences based on demographic variables and/or geographic location, leading to patients with similar backgrounds and functional limitations being referred to different types of rehabilitation. Given that different rehabilitation options have different outcomes, this variation in referral preferences may lead to suboptimal rehabilitation outcomes for some stroke patients.^[5]^

## 2 Methods

### 2.1 Study design

This study was approved by the Institutional Review Board of Columbia University Medical Center. Three of the authors administered a survey to physiatrists attending the American Academy of Physical Medicine and Rehabilitation (AAPM&R) Annual Assembly (November 13–16, 2014; San Diego, CA). Participants were approached by investigators in common areas of the conference (e.g., coffee area, corridors) and asked to complete the survey. This sampling method was selected to avoid the low response rates typically seen with surveys distributed electronically or by mail. Participants were given the option of completing the survey on paper or on a computer tablet (via Survey Monkey).

The survey collected basic demographic information about respondents and presented 5 fictional poststroke cases. Demographic data included trainee status, board certification, academic affiliation, extent of active involvement in the care of stroke patients, and practice affiliation(s). It also included state of practice, age, and number of years in practice. The 5 case vignettes included information about medical, social, and functional domains as seen in Table 1. The cases were designed to represent commonly encountered scenarios in clinical practice presenting a range of rehabilitation, medical, and social needs. For each case, respondents were asked to indicate their first and second choice of postacute rehabilitation setting, and how strongly they felt regarding the assigned setting. They were also presented with 15 variables and asked to rate the influence of these factors on the referral decision on a scale of 1 to 10 (1 indicated the lowest level of influence and 10 indicated the highest level of influence). The vignettes assumed that each patient was in a stroke unit after having suffered an acute stroke and that the referral decision was made within a week after stroke. The entire survey is available upon request.

**Table 1:**
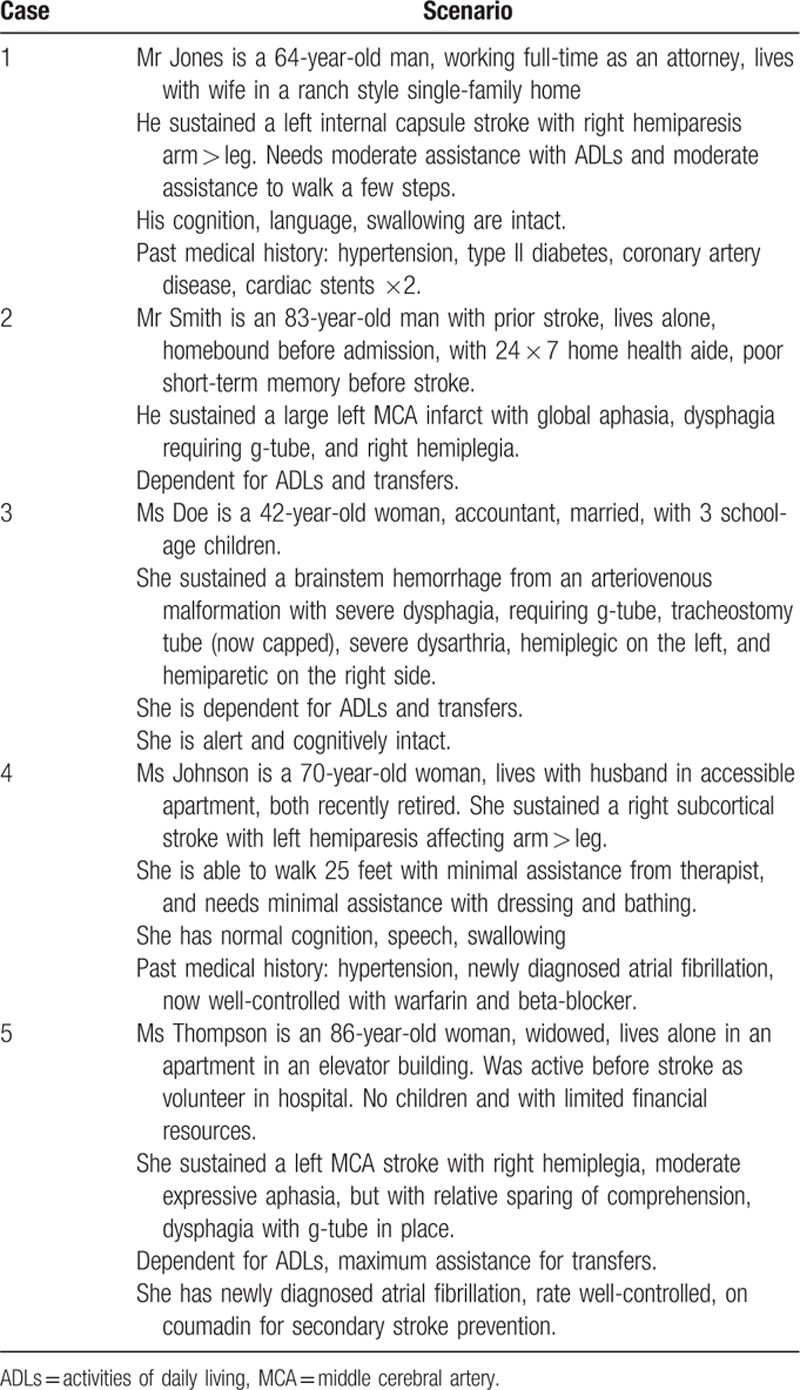
Case scenarios provided in survey for disposition referral determination.

### 2.2 Study participants

Participants (n = 86) included residents, fellows, and attending physicians out of the possible reported 2638 conference attendees.

### 2.3 Statistical analysis

Demographic variables were collapsed for analysis, including location of practice (grouped into regions using the US Census Bureau categories), age (grouped into 40 and above, or below 40, based on the mean age of our respondents of 39), and years in practice (grouped into 10 years or more, or fewer than 10 years). Statistical analysis was completed using SPSS (IBM Corp. Released 2014, IBM SPSS Statistics for Windows, Version 23.0. Armonk, NY: IBM Corp.) Bivariate analysis using the chi-square test was used to assess the relationship between referral preferences and all of the demographic variables. Subsequently, the Cramér V was used to further determine the strength of association between referral pattern and practice affiliation (please refer to supplemental content for complete data set).

## 3 Results

Eighty-six surveys were collected over a 3-day period. Demographic data for the 86 respondents are described in Table 2. Some participants did not complete all items in the survey; the data reported for each item reflect only the actual respondents for that item. Bivariate analysis (using chi-square) showed no statistically significant relationship between any of the demographic variables and poststroke rehabilitation preference for any of the cases.

**Table 2:**
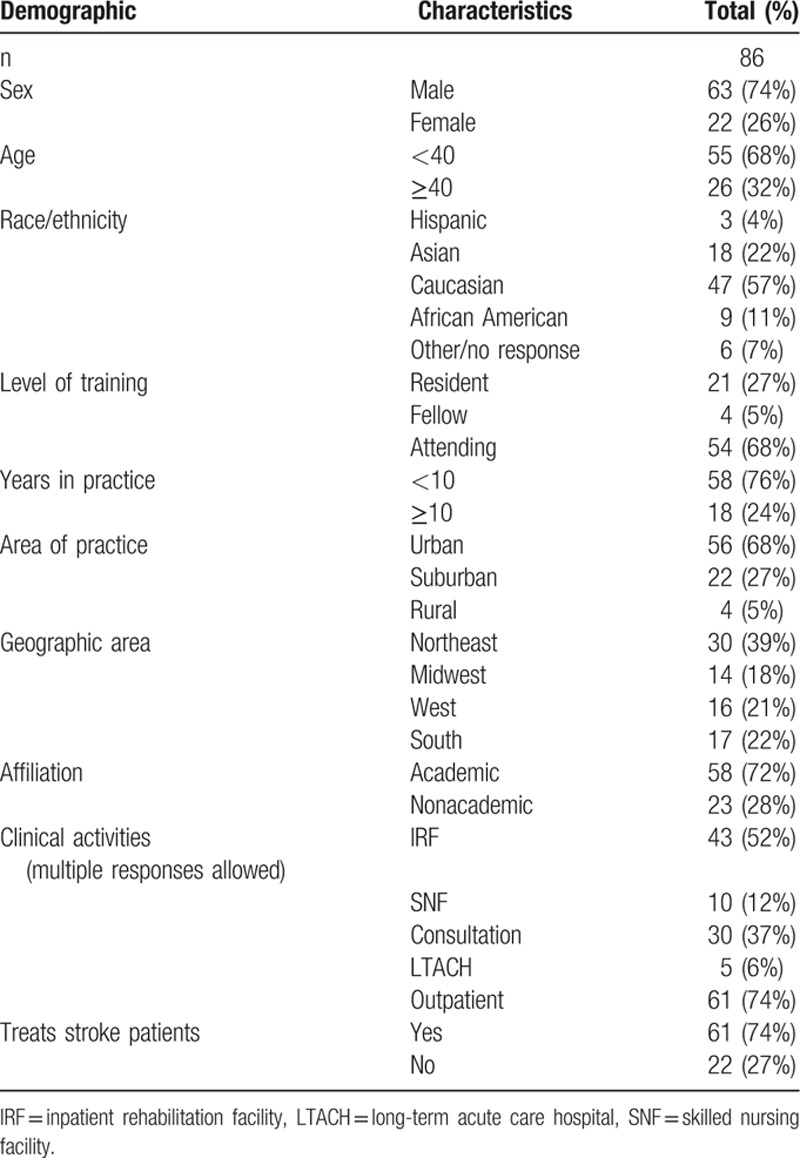
Demographic characteristics of participants.

As shown in Table 3, case 1 showed the least variation in discharge recommendation: 93% of the respondents selected IRF as the most appropriate discharge destination. Similarly, in case 3, 82% selected IRF. The remainder of the cases had more variation. In case 2, 44% of respondents selected SNF, 25% selected IRF, and 21% selected LTACH. In case 4, 60% selected IRF and 30% selected home (with services or outpatient care). Finally, in case 5, 56% of respondents selected IRF, 27% selected SNF, and 15% selected LTACH.

**Table 3:**
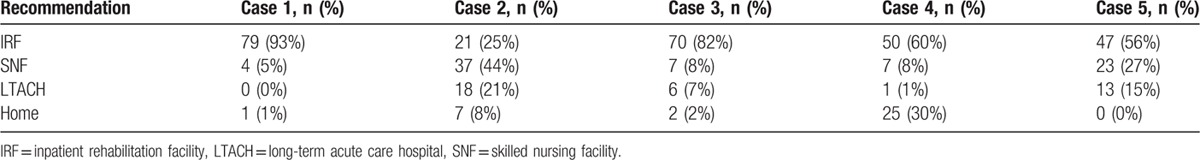
First choice of discharge destination by case scenario.

**Figure 1:**
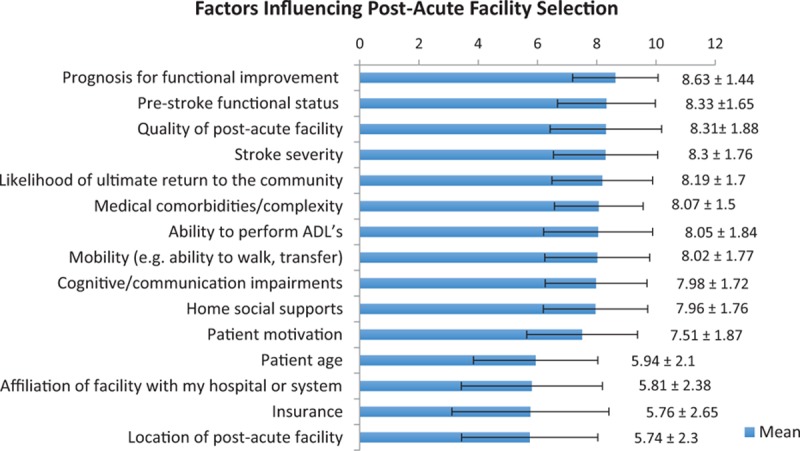
Influencing factors.

Figure 1 shows the respondents’ assessment of various factors’ degree of influence on their referral decisions. Among the 15 variables, the mean ratings ranged from 5 to 8, and the median ratings ranged from 5 to 9. The prognosis for functional outcome and quality of postacute facility had the highest mean influence ratings (8.63 and 8.31, respectively), whereas location of postacute facility and insurance had the lowest mean influence ratings (5.74 and 5.76, respectively). Quality of postacute facility, stroke severity, prestroke functional status, and prognosis for functional improvement had the highest median influence ratings (9) and insurance had the lowest median influence rating (5).

## 4 Discussion

To the best of our knowledge, this is the first published study examining postacute stroke rehabilitation referral preferences by physiatrists in the United States. Although physiatrists are not always part of the referral decision in every medical institution, we believe that their participation is an important component in optimal patient outcomes. Our results indicate that there is substantial practice variation for certain case vignettes, perhaps representing patients whose characteristics overlap “ideal” patient characteristics for 2 or more levels of care. We did not, however, find any regional practice differences in this study, nor did we find other demographic variables that were associated with referral preferences. Whereas we do not have any “gold standard” to determine the appropriate discharge destination for the patients represented in the vignettes, the pattern of referral by respondents seems to favor referral to an IRF to a notable degree.

Medicare data have shown that the largest number of stroke patients in the United States receive rehabilitation care in a SNF after discharge (32%), followed by IRF (22%) and home health care (15%).^[1,18]^ In this study, referral to IRF was generally favored over other options, showing that the majority of physiatrists refer poststroke patients to IRF. This may reflect greater familiarity with IRF care, given the key role that IRFs play in physiatry training and practice. It may also reflect an explicit preference for IRF among physiatrists because of belief in superior care or superior functional outcomes for persons with stroke. It would be interesting to conduct a similar survey of other rehabilitation professionals, such as physical and occupational therapists, whose training and practice may be less IRF-centric.

Future study in this field has the potential to lead to standardized assessment tools and protocols for healthcare practitioners who make poststroke referral decisions. Such tools may reduce undesirable variation in care, and help provide the most appropriate level of rehabilitation to stroke survivors.

### 4.1 Study limitations

This study has several important limitations. As would be expected in a small survey study, the respondent sample does not represent physiatrists in general. The survey was administered only to a convenience sample of physiatrists with the time, availability, and financial resources to attend the AAPM&R Annual Assembly. Both academic physiatrists and young, male physiatrists who were relatively new to the field were over-represented in the sample. Also, as with any survey study, there is inherent recall bias, and responses may reflect more of an idealized version of practice preferences rather than actual experience. Since some individuals chose not to participate in the survey, and some responders did not complete every question, the data are limited by nonresponder bias. Additionally, whereas the case vignettes were designed to highlight key patient variables that may influence referral decisions, and whereas the respondents rated the importance of different variables in general as seen in Fig. 1, these data do not provide any information about which variable(s) was influential in a given case.

Future research should further clarify which patient factors are most influential for physiatrists (and other providers) as they make referral decisions. Additionally, more information is needed about which patient variables are most predictive of success in different rehabilitation settings. Once these pieces are in place, guidelines can be created to guide practitioners in these decisions.

## 5 Conclusions

Physiatrists’ referral preferences did not vary with any identified practitioner variables; referral preferences only varied significantly by case. Further study is needed to determine the patient factors that most influence referral decisions, and to determine which factors should be used to guide referral decisions for optimal patient outcomes.

## Acknowledgment

The authors gratefully thank Lauri Bishop DPT, for her advice regarding data analysis.
